# Individual, employment and psychosocial factors influencing walking to work: Implications for intervention design

**DOI:** 10.1371/journal.pone.0171374

**Published:** 2017-02-09

**Authors:** Emma J. Adams, Dale W. Esliger, Ian M. Taylor, Lauren B. Sherar

**Affiliations:** National Centre for Sport and Exercise Medicine, School of Sport, Exercise and Health Sciences, Loughborough University, Loughborough, United Kingdom; TNO, NETHERLANDS

## Abstract

**Background:**

Promoting walking for the journey to and from work (commuter walking) is a potential strategy for increasing physical activity. Understanding the factors influencing commuter walking is important for identifying target groups and designing effective interventions. This study aimed to examine individual, employment-related and psychosocial factors associated with commuter walking and to discuss the implications for targeting and future design of interventions.

**Methods:**

1,544 employees completed a baseline survey as part of the ‘Walking Works’ intervention project (33.4% male; 36.3% aged <30 years). Multivariate logistic regression was used to examine the associations of individual (age, ethnic group, educational qualifications, number of children <16 and car ownership), employment-related (distance lived from work, free car parking at work, working hours, working pattern and occupation) and psychosocial factors (perceived behavioural control, intention, social norms and social support from work colleagues) with commuter walking.

**Results:**

Almost half of respondents (n = 587, 49%) were classified as commuter walkers. Those who were aged <30 years, did not have a car, had no free car parking at work, were confident of including some walking or intended to walk to or from work on a regular basis, and had support from colleagues for walking were more likely to be commuter walkers. Those who perceived they lived too far away from work to walk, thought walking was less convenient than using a car for commuting, did not have time to walk, needed a car for work or had always travelled the same way were less likely to be commuter walkers.

**Conclusions:**

A number of individual, employment-related and psychosocial factors were associated with commuter walking. Target groups for interventions to promote walking to and from work may include those in older age groups and those who own or have access to a car. Multi-level interventions targeting individual level behaviour change, social support within the workplace and organisational level travel policies may be required in order to promote commuter walking.

## Introduction

Participating in regular physical activity is known to benefit health [1–3]. However, 33% of males and 45% of females in England do not meet current physical activity recommendations [4]. As a result, there is an increasing burden on public health and the economy due to the increased prevalence of chronic diseases associated with low levels of physical activity [5–7]. Promoting walking is one potential strategy to increase physical activity levels. It requires no special skills or equipment, has been described as the perfect exercise for most adults [8] and is known to have health benefits [9–14]. Furthermore, when walking is used for transport purposes (to travel from one place to another) it may reduce transport costs, help save money, improve the environment by reducing pollution, reduce traffic congestion, improve road safety and lead to social benefits such as increased social interactions and sense of community [1,15].

Currently, it is estimated that only between 9% and 10% of adults in England walk for their daily commute with 63% to 67% travelling by car, either as a driver or as a passenger [16,17]. Increasing the proportion of adults who walk to and from work is one approach which has the potential to contribute to higher levels of physical activity [18–20]. In one study, total weekday physical activity was found to be 45% higher in participants who walked to work compared to those who commuted by car, and moderate-to-vigorous physical activity (MVPA) almost 60% higher, in a sample of adults employed in England [18]. Walking to and from work has also been shown to lead to improvements in health [21–25] as well as conferring the wider benefits outlined above. A high proportion (74.5%) of the adult population in England is in employment [26] and 72.3% (66.0% males; 80.0% females) travel to and from the same place of work each day [16]. Encouraging walking to and from work either for the entire journey or for part of the journey in combination with other modes of transport (commuter walking), may therefore help to increase physical activity and improve health in a large proportion of the population. In order to develop effective interventions, a better understanding of the factors influencing commuter walking is required.

Due to the large range of potential influences on behaviour, theoretical models are often used to identify and understand relationships between different factors. Socio-ecological models identify multiple levels of influence on behaviour including individual, intra- and inter-personal, organisational, environmental and policy-related factors [27–30] whilst theories, such as the theory of planned behaviour (TPB) [31], are used to assess specific psychological influences. The TPB is the most commonly used in assessing psychological factors influencing active travel [32]. In addition, studying behaviour- and context-specific individual, psychological, social and environmental characteristics as part of more comprehensive ecological models has been identified as being important [33]. Whilst some studies have assessed the factors influencing the specific behaviour of active commuting (walking and cycling combined) [34–38], it has been recommended that walking and cycling should be studied separately given their functional differences [39].

Only a small number of studies have examined the factors influencing the specific behaviour of commuter walking [23,40–42]. To date, only two studies have assessed the influence of employment-related factors such as working hours and occupation [23,40] and none have investigated working pattern (e.g. shift work or normal office hours). Further evidence is also needed to understand the relationship between perceived barriers and psychological factors such as attitudes, perceived behavioural control and intention with commuter walking. In addition, the influence of social norms in relation to commuter walking (whether an individual perceives that most of their colleagues walk to and from work) and the role of the social environment at work (support received from colleagues for commuter walking) have been relatively unexplored. Understanding which of these factors are associated with commuter walking is important for identifying target groups and for designing effective interventions. The aim of this study was to investigate the associations among individual, employment-related and psychosocial factors and commuter walking and to discuss the implications for the future design and targeting of interventions.

## Methods

The ‘Walking Works’ intervention project engaged with five employers in England based in the North East, Yorkshire, East Midlands, West Midlands and London. As part of the intervention, volunteer ‘walking champions’ (n = 8) were recruited who developed and delivered walking initiatives in their workplaces. The walking champions were provided with resources (such as materials to promote a national walk to work campaign and pedometer challenges) to encourage people to walk for all or some of their journey to work, as well as to walk more during the working day. Full details of the intervention are reported elsewhere [43]. Data used in this study were collected in the baseline survey which was conducted between December 2009 and June 2010. All employees in each of the five workplaces (total N = 5,512) were sent an invitation to complete the survey and 1,544 employees responded (28% response rate). Ethical approval for this study was obtained from Loughborough University Ethical Advisory Committee (reference R09-P121). All respondents gave written informed consent to participate in the survey.

### Measures

#### Commuter walking

Respondents were asked to report the number of minutes they spent walking to and from work separately for each day during the last week (including week and weekend days). Total number of minutes spent walking to and from work in the last week were computed by summing the number of minutes spent walking to and from work for each day. As the data were positively skewed, a binary variable was computed for commuter walking. Respondents were classified as ‘commuter non-walkers’ if they did 0 minutes of walking to and from work in the last week, or did not report any trips of at least 10 minutes (as Government recommendations suggest bouts of 10 minutes or more MVPA are needed to benefit health [1]). All other respondents with data for total minutes spent walking to and from work in the last week were classified as ‘commuter walkers’.

#### Physical activity

Overall physical activity was assessed using the short International Physical Activity Questionnaire (IPAQ) which has been shown to have acceptable reliability and validity [44]. Total minutes per week of vigorous intensity physical activity, moderate intensity physical activity and walking were computed and these were summed to give the total minutes of MVPA in a usual week (7 days). In order to assess physical activity levels at work, respondents reported whether their work was mainly completed sitting, standing or was manual or heavy manual work (taken from the European Prospective Investigation in Cancer and Nutrition questionnaire (EPIC) Medical Research Council, Cambridge). Responses were collapsed so those who spent most of their time sitting were compared to those with non-sitting occupations (standing, manual or heavy manual work).

#### Psychosocial factors

Respondents were asked to what extent they agreed with four statements to assess selected constructs from the TPB [31]: 1) I believe everyone should walk for part or all of their journey to and from work (attitude); 2) I am confident that I can include some walking as part of my journey to or from work on most days (perceived behavioural control); 3) I intend to walk for all or part of my journey to or from work on a regular basis in the next few months (intention) and 4) Most of my colleagues walk for some, or all, of their journey to or from work (social norms). Response options to all statements were on a 4-point Likert scale from strongly disagree to strongly agree. Responses to each item were collapsed so that those who ‘strongly disagreed’ or ‘disagreed’ were compared to those who ‘agreed’ or ‘strongly agreed’, a similar approach has been used for assessing single-item measures of environmental factors [42].

Perceived social support from work colleagues for walking to and from work was assessed by asking: during the past month, how much have your work colleagues encouraged you to walk for some, or all, of your journey to or from work? Response options were on a five point Likert scale: never, rarely, sometimes, often or very often. Responses were collapsed so that those who perceived they had support ‘never’ or ‘rarely’ (disagree) were compared to those who perceived they had support ‘sometimes’, ‘often’ or ‘very often’ (agree). A sensitivity analysis was conducted in which ‘never’, ‘rarely’ and ‘sometimes’ (disagree) were compared to those who perceived they had support ‘often’ or ‘very often’ (agree) and this made no difference to the overall results. Finally, perceived barriers to walking to work were assessed by asking respondents to indicate the main reasons they would give for not walking for some, or all, of the journey to/from work from a pre-defined list of 17 statements. A tick was used to indicate agreement with the statement and the top ten most frequently reported barriers (n = >100 responses for each barrier) were included in this study. Those who considered the item to be a barrier were compared to those who did not.

#### Individual and employment-related factors

Respondents reported their gender, age, ethnic group, highest educational qualification, number of children under 16 and car ownership (or access to a car) as well as the distance they lived from work, the availability of free car parking at work, work hours (full-time or part-time), working pattern (e.g. regular day time hours or shift pattern) and their occupation (selected from: senior managers or directors, middle or junior managers, traditional professional occupations, modern professional occupations, clerical and administrative occupations, technical and craft occupations, or semi-routine manual and service occupations).

### Analyses

Respondents were included in the analysis if they provided data for time spent walking to or from work for at least one day of the week. Descriptive data were summarised using percentages. Chi square tests were used to assess differences between those included in the analysis and those excluded. Univariate associations of individual, employment-related and psychosocial factors were examined using logistic regression analysis with commuter non-walkers as the reference category. Variables that were significant (p<0.05) in univariate analysis were carried forward into the multivariate analysis. A series of multivariate logistic regression models were built to calculate the odds ratios of commuter walking (none/some) associated with individual, employment and psychosocial factors. These were entered in blocks (Model A: individual and employment-related factors; Model B: model A with perceived barriers added; Model C: model B with psychosocial factors added) to enable the contribution of each to be assessed using the R^2^ statistic [45]. To maximise the predictive power of the fitted model, backwards stepwise regression was used to identify variables (from those which were significant in univariate analysis) most strongly associated with the outcome measure. Data were analysed in SPSS Statistics (version 22.0) (IBM SPSS Inc, Armonk, New York).

## Results

Overall, 1,189 (77%) survey respondents were included in the analysis. Compared to those included in the analysis, a higher proportion of excluded respondents were female (80.5% vs. 65.6%, p = <0.001), more excluded respondents were aged 30 or older (75.5% vs. 63.7%, p = 0.01), a higher proportion had cars (94.8% vs. 84.8%, p<0.001), fewer employees spent most of their time at work sitting (86.2% vs. 93.1%, p = <0.001) and a higher proportion reported having free car parking at work (80.3% vs. 60.1%, p = <0.001). There were no significant differences on other demographic and work-related characteristics. Table 1 shows the individual and employment-related characteristics of all respondents included in the analyses (33.4% male; 36.3% aged <30 years). Almost half of respondents (n = 587, 49%) were classified as commuter walkers. Median time spent walking to and from work in the last week for commuter walkers was 120 minutes (Interquartile range (IQR) 80–200) and the median time spent walking per trip was 20 minutes (IQR 10–25). Univariate associations of individual and employment-related factors, perceived barriers and other psychosocial factors with commuter walking are shown in S1 and S2 Tables.

**Table 1:** Sample characteristics for commuter non-walkers, commuter walkers and overall.

Characteristic	Commuter non-walkers		Commuter walkers		Overall	
	n = 602		n = 587		n = 1189	
	n^a^	%	n^a^	%	n^a^	%
**Gender**						
Female	368	64.6	362	66.7	730	65.6
**Age (years)**						
<30	158	29.8	222	43.1	380	36.3
30–44	211	39.7	178	34.6	389	37.2
≥45	162	30.5	115	22.3	277	26.5
**Ethnic group**						
White	535	96.9	489	93.3	1024	95.2
**Education**						
Higher degree/degree	292	51.8	234	43.4	526	47.7
GCE ‘A’ Level	145	25.7	149	27.6	294	26.7
GCSE Grades A to C	104	18.4	138	25.6	242	21.9
No formal qualification	23	4.1	18	3.3	41	3.7
**Children under 16**						
Yes	182	30.2	138	23.5	320	26.9
**Car ownership/access**						
Yes	544	96.3	390	72.6	934	84.8
**Physical activity**^b^						
Total MVPA (mean minutes ±SD in a usual week)	594	568.2 ±511.2	586	718.9 ±554.3	1189	643.0 ±538.1
**Physical activity at work**						
Sitting occupation	542	91.1	552	95.2	1094	93.1
Non-sitting occupation	53	8.9	28	4.8	81	6.9
**Distance live from work**						
<2 miles	66	11.0	195	33.7	261	22.2
2.1–5 miles	155	25.9	175	30.2	330	28.0
5.1–10 miles	177	29.6	101	17.4	278	23.6
>10 miles	200	33.4	108	18.7	308	26.2
**Free car parking at work**						
Yes	423	74.9	235	44.4	658	60.1
**Working hours**						
Full-time	441	77.5	429	78.4	870	78.0
**Working pattern**						
Regular day time hours	527	92.9	466	85.8	993	89.5
Shift patterns	40	7.1	77	14.2	117	10.5
**Occupation**						
Senior or Middle Manager	110	19.3	78	14.2	188	16.8
Professional occupation	173	30.4	103	18.8	276	24.7
Clerical or manual	287	50.4	367	67.0	654	58.5
**Organisation**^c^						
A	43	7.1	30	5.1	73	6.1
B	220	36.5	312	53.2	532	44.7
C	143	23.8	70	11.9	213	17.9
D	170	28.2	146	24.9	316	26.6
E	26	4.3	29	4.9	55	4.6

^a^Numbers do not sum to total due to missing responses

^b^Assessed using the short form of the International Physical Activity Questionnaire [44]

^c^Organisations: A = Further Education Institution, North East; B = Private Organisation, East Midlands; C = NHS Organisation, Yorkshire; D = County Council, West Midlands; E = Higher Education Institution, London

In multivariate models, a range of employment-related factors, perceived barriers and psychosocial factors were significantly associated with commuter walking (Table 2). In the first model (Model A), car ownership, distance to work and not having free car parking at work were significantly associated with commuter walking. The pseudo-R^2^ value indicated that individual and employment-related factors explained 36.5% of the variance in commuter walking. When perceived barriers were added to the model (Model B), car ownership, distance to work and not having free car parking at work remained significantly associated with commuter walking. In addition, respondents aged <30 years were significantly more likely to walk to work and those who perceived they lived too far from work, thought walking was less convenient than using a car or took too long, or perceived that they did not have time or needed a car for work, were significantly less likely to walk to work. The total variance in commuter walking that could be explained when perceived barriers were added into the model increased by 11.7% to 48.2%.

**Table 2:** Multivariate regression models for likelihood of commuter walking.

	Model A		Model B		Model C	
	OR (CI)^a^	p-value	OR (CI)^a^	p-value	OR (CI)^a^	p-value
**Individual factors**^b^						
Age (reference: ≥30 years)						
<30 years	1.26 (0.90, 1.77)	0.180	1.67 (1.14, 2.43)	**0**.**008**	1.82 (1.09, 3.06)	**0.023**
Own/access to a car (reference: Yes)						
No	8.27 (4.66, 14.65)	**<0.001**	5.20 (2.84, 9.54)	**<0.001**	5.29 (2.23, 12.52)	**<0.001**
**Employment-related factors**						
Work-related physical activity (reference: Non-sitting)						
Sitting	1.23 (0.64, 2.38)	0.531	1.54 (0.76, 3.10)	0.228	1.47 (0.56, 3.84)	0.429
Distance lived from work (reference: >5 miles)						
≤2 miles	4.58 (3.03, 6.92)	**<0.001**	2.65 (1.53, 4.61)	**0.001**	1.28 (0.59, 2.76)	0.534
2.1–5 miles	1.88 (1.32, 2.67)	**<0.001**	1.72 (1.10, 2.67)	**0.016**	1.48 (0.81, 2.71)	0.200
Free car parking at work (reference: Yes)						
No	4.55 (3.27, 6.32)	**<0.001**	3.96 (2.78, 5.65)	**<0.001**	3.00 (1.83, 4.89)	**<0.001**
Occupation (reference: Manager/professional)						
Clerical or administrative	1.25 (0.86, 1.82)	0.238	1.29 (0.85, 1.97)	0.237	1.10 (0.62, 1.94)	0.750
Work pattern (reference: Regular day time hours)						
Shift patterns	1.30 (0.76, 2.23)	0.334	1.34 (0.74, 2.43)	0.331	1.08 (0.44, 2.63)	0.868
**Perceived barriers**^c^						
I live too far away from work	-	-	0.57 (0.37, 0.87)	**0.009**	0.74 (0.40, 1.36)	0.330
Less convenient than using a car	-	-	0.29 (0.19, 0.43)	**<0.001**	0.33 (0.19, 0.58)	**<0.001**
It takes too long	-	-	0.62 (0.42, 0.90)	**0.012**	0.95 (0.56, 1.60)	0.835
I don’t have time	-	-	0.67 (0.45, 0.99)	**0.045**	0.57 (0.33, 0.97)	**0.037**
I need a car to do my job	-	-	0.51 (0.31, 0.83)	**0.007**	0.61 (0.31, 1.18)	0.139
I have too much to carry	-	-	1.17 (0.74, 1.85)	0.508	1.37 (0.72, 2.60)	0.334
Need to do other activities on the way	-	-	1.38 (0.85, 2.24)	0.191	1.38 (0.72, 2.61)	0.331
Need to drop off/collect children	-	-	0.73 (0.43, 1.26)	0.256	0.93 (0.45, 1.95)	0.856
I do lots of other activity/sport	-	-	0.76 (0.47, 1.23)	0.262	0.62, (0.33, 1.17)	0.139
I’ve always travelled the same way	-	-	0.57 (0.32, 1.02)	0.057	0.40 (0.19, 0.88)	**0.022**
**Psychosocial factors**						
Attitude^d^	-	-	-	-	1.48 (0.92, 2.38)	0.106
Perceived behavioural control^d^	-	-	-	-	4.57 (2.58, 8.11)	**<0.001**
Intention^d^	-	-	-	-	3.51 (1.95, 6.32)	**<0.001**
Social norms^d^	-	-	-	-	0.84 (0.51, 1.40)	0.510
Social support from colleagues^e^	-	-	-	-	1.98 (0.97, 4.03)	0.059
Nagelkerke R^2^	.365		.482		.654	

^a^OR (CI) = odds ratio (95% confidence interval)

^b^Ethnic group, education, children under 16 and organisation were also included; findings were non-significant in all models

^c^The reference category is no (item is not considered a barrier)

^d^The reference category is strongly disagree or disagree

^e^The reference category is disagree

In the final adjusted model, which included individual factors, employment-related factors, perceived barriers and psychosocial factors (Model C), being aged <30 years and not owning or having access to a car were significantly associated with the likelihood of being a commuter walker. Respondents were three times (odds ratio (OR) 3.00, 95% confidence interval (95% CI) 1.83 to 4.89) more likely to walk to work if there was no free car parking at work, and around four times more likely to walk to work if they were confident they could walk to work on most days or if they intended to walk to work on a regular basis (OR 4.57, 95% CI 2.58 to 8.11 and OR 3.51, 95% CI 1.95 to 6.32 respectively). In addition, employees who perceived walking to work to be less convenient than using a car, perceived they did not have time or have always travelled to work the same way were significantly less likely to be commuter walkers. Including individual factors, employment-related factors, perceived barriers and psychosocial factors increased the variance in commuter walking which could be explained by the model to 65.4%.

The multivariate regression models described above aimed to explore the associations between different variables and commuter walking and assess how much the blocks of variables contributed to relationships. A stepwise model was then used to identify the best combination of variables most strongly associated with commuter walking (Table 3). The variables remaining in the final stepwise model included those which were significant in Model C. A perception of living too far away from work to walk and needing a car for work were also retained in the stepwise model. These variables were negatively and significantly associated with commuter walking. In the stepwise regression the variables remaining in the model explained 63.9% of the variance in commuter walking.

**Table 3:** Stepwise regression model for individual, employment-related and psychosocial factors and likelihood of commuter walking.

	Commuter walking	
	OR (CI)^a^	p-value
**Individual factors**		
Age (reference: ≥30 years)		
<30 years	1.72 (1.09, 2.71)	**0.019**
Own/access to a car (reference: Yes)		
No	5.63 (2.50, 12.71)	**<0.001**
**Employment-related factors**		
Free car parking at work (reference: Yes)		
No	2.82 (1.80, 4.43)	**<0.001**
**Perceived barriers**^b^		
I live too far away from work	0.62 (0.39, 0.99)	**0.044**
Less convenient than using a car	0.37 (0.23, 0.60)	**<0.001**
I don’t have time	0.60 (0.38, 0.97)	**0.035**
I need a car to do my job	0.49 (0.28, 0.84)	**0.010**
I’ve always travelled the same way	0.44 (0.22, 0.91)	**0.026**
**Psychosocial factors**		
Perceived behavioural control^c^	4.26 (2.47, 7.35)	**<0.001**
Intention^c^	3.63 (2.08, 6.37)	**<0.001**
Social support from colleagues^d^	2.32 (1.17, 4.60)	**0.016**
Nagelkerke R^2^	.639	

^a^OR (CI) = odds ratio (95% confidence interval)

^b^The reference category is no (item is not considered a barrier)

^c^The reference category is strongly disagree or disagree

^d^The reference category is disagree

## Discussion

A number of individual, employment-related and psychosocial factors were associated with commuter walking. Individuals aged <30 years and those not owning a car were more likely to be commuter walkers in almost all models; similar findings have been reported elsewhere. For example, using a nationally representative survey in the United Kingdom with a sample of 20,458 adults, participants aged 16–29 years were found to be more likely to walk to work than older participants [23]. In a self-report staff travel survey undertaken at a University in England, which was completed by 2,829 employees, it was found that those aged under 35 years were more likely to walk to work [46]. In relation to car ownership, in a sample of 1,142 employees who completed a self-report survey as part of a study in Cambridge (United Kingdom), not having access to a car, along with living a short distance from work, was found to be positively associated with walking to work [41]. This suggests that in practice, target groups for interventions to promote walking to work may include those in older age groups and those who own or have access to a car.

Previous studies have shown individuals are more likely to walk as part of their journey to work if they live within a short distance (<3km) of their workplace [41]. In this study, we examined self-reported distance from work and perceptions of the distance lived from work by assessing the barrier for walking “I live too far away from work”. In the stepwise model, only the perception of living too far from work was significantly associated with commuter walking. This suggests perceived distance may be more informative in explaining commuter walking behaviour than actual distance and warrants further investigation. Addressing the perceived barrier of living too far away from work in interventions may be important in terms of helping those who live some distance away from work to understand how they can incorporate walking as part of their journey in combination with another form of transport (rather than walking for the whole journey).

Lack of free car parking at work was strongly associated with commuter walking and this also supports previous findings [47]. Employers should therefore be encouraged to implement workplace travel policies which discourage car use by including a charge for parking. This may help to reduce the use of the car as a mode of transport to travel to work and thereby promote walking. There is some evidence to suggest these policies can be effective in reducing car use and increasing walking levels [46,48,49]. However, further robust evaluation of the impact is needed specifically for commuter walking behaviour (rather than combined with cycling).

Psychological factors such as perceived behavioural control for walking to work (defined as the perceived ease or difficulty of performing the behaviour) [31] and intention to walk to work were also strongly associated with commuter walking. In a recent review of behaviour change techniques for promoting walking and cycling, prompt self-monitoring of behaviour (i.e. the person is asked to keep a record of a specified behaviour), which has been shown to increase efficacy beliefs and reduce perceived barriers, and prompt intention formation (i.e. encourage the person to decide to act or set a general goal) were included in over half of the interventions that reported statistically significant changes in walking and cycling [50]. This study adds some support for the potential inclusion of these approaches in future interventions to promote walking.

In relation to specific barriers for commuter walking, the findings showed that employees who perceive walking to work is “less convenient than using a car” and that “a car is needed to carry out their job” are less likely to be commuter walkers. This suggests that interventions may need to focus on making car use less convenient (e.g. by moving car parking off-site or further away from workplace buildings) and by providing pool cars such that individual employees do not need to bring their own car to work. Again, these might form part of a more comprehensive workplace travel strategy. The finding that “I’ve always travelled the same way” was negatively associated with commuter walking suggests that habit (behaviour that has been performed routinely or repetitively and occurs automatically rather than intentionally [51]) may be important in influencing choice of travel mode. The potential importance of habit and its role in travel behaviour has been reported elsewhere [32]. Future interventions may therefore need to consider targeting habit reduction in car users as an approach to shifting individuals towards using other modes such as walking. There is currently limited evidence relating to the efficacy of behavioural interventions to reduce car use and further research is needed to investigate evidence- and theory-based interventions using more robust study designs [52].

A number of studies have examined associations between social support from family or friends and active travel [32]. However, this is the first study the authors of this paper are aware of to report findings on social support from colleagues for walking to work, which was found to be strongly associated with commuter walking. Interventions which use a buddy scheme, or walking “champions” who encourage other employees to walk to and from work, might provide a source of support for commuter walking. An initial pilot study has demonstrated that using trained walking promoters to support walking to work is feasible [53].

Understanding commuter walking is complex due to the wide range of factors which influence the behaviour. The findings above suggest interventions should target specific sub-groups of the population. They also provide some direction as to the type and content of interventions that might be delivered in order to promote commuter walking to address the wide range of factors. Individual interventions using specific behaviour change techniques, combined with social support from work colleagues and organisational travel policies which discourage car use, may be needed to support behaviour change for commuter walking. Future research should investigate these types of multi-level interventions.

### Strengths and limitations

The strengths of this study included examination of a wide range of potential factors that may influence walking to and from work to try and understand behaviour- and context-specific correlates. This is one of few studies to investigate the associations between individual, employment-related and psychosocial factors with the specific behaviour of commuter walking. Other studies have often combined walking with cycling to explore influences on overall active modes of commuting. This has made it difficult to assess specific influences on walking which may differ from those related to cycling. Data were collected over a six month period between December and June which may help to take into account seasonal variations in travel behaviour.

The limitations of this study included relying on self-report measures which may have resulted in both measurement error and bias [54]. Both commuter walkers and commuter non-walkers reported high levels of total MVPA which on average exceeded current Government recommendations. These observations may be due to over-reporting of physical activity levels resulting from using the short form of the IPAQ. This questionnaire has previously been reported to overestimate physical activity levels by on average 106% (range 36–173%) when compared to accelerometer-measured physical activity [55]. Similarly, a relatively high proportion of respondents in this study were classified as commuter walkers. This may be because, in contrast to national surveys, this study included walking as part of multi-modal trips as well as when it used as a single mode of travel. There could also have been over-reporting of walking levels. The intensity of commuter walking was not assessed in the survey, therefore, it is not known whether the walking undertaken was sufficient to be classified as MVPA. Future studies would benefit from using objective measures such as accelerometers to measure physical activity, time spent walking on the daily commute and the level of intensity of the walking undertaken. The high levels of physical activity reported and differences in characteristics between the samples included and excluded from the analyses suggest the sample included in this study may not have been truly representative of the population leading to selection bias. Furthermore, the low response rate to the survey may also have led to bias.

Not all the components of the TPB were assessed in this study and only single item questions were used to measure the constructs. Other psychological constructs and factors from broader ecological models, such as the built environment and transport infrastructure in and around neighbourhoods and workplaces, may also be important in influencing commuter walking and remain important areas for future study. Finally, due to the cross sectional nature of this study, inferences regarding causal relationships between individual, employment-related and psychosocial factors and commuter walking cannot be made.

## Conclusion

A number of individual, employment-related and psychosocial factors were associated with commuter walking. In particular, age, car ownership, availability of free car parking, perceived barriers relating to distance lived from work, convenience, time, car use for work and habit, as well as confidence, intention and social support from colleagues may influence whether an individual walks for all, or as part of, their daily commute. Together these findings help us to further understand the factors associated with commuter walking which should be taken into consideration in the targeting and design of future interventions. Multi-level interventions targeting individual level behaviour change, social support within the workplace and organisational level travel policies may be required in order to promote commuter walking.

## Supporting information

S1 Table Univariate associations for individual and employment-related factors and likelihood of commuter walking. (PDF)

S2 Table Univariate associations for perceived barriers, psychosocial factors and likelihood of commuter walking. (PDF)
