# Supplementary material for: Individual, employment and psychosocial factors influencing walking to work: Implications for intervention design
Source: PLoS One. 2017 Feb 9;12(2):e0171374. doi: 10.1371/journal.pone.0171374 (PMC5300108; doi:10.1371/journal.pone.0171374)
Supplement: S1 Table — (PDF) [file pone.0171374.s001.pdf]

Table S1. Univariate associations for individual and employment-related factors and likelihood of commuter walking

	OR (CI)[a]	p-value
**Individual factors**		
Sex (reference: Male)		
Female	1.10 (0.86, 1.41)	0.460
Age (reference: ≥30 years)		
<30 years	1.79 (1.39, 2.31)	**<0.001**
Ethnic group (reference: White)		
Non-white	2.25 (1.25, 4.07)	**0.007**
Education (reference: Degree or higher degree)		
No degree	1.40 (1.10, 1.77)	**0.006**
Children under 16 years old (reference: Yes)		
No	1.41 (1.09, 1.83)	**0.009**
Own/access to a car (reference: Yes)		
No	9.76 (6.07, 15.71)	**<0.001**
**Employment-related factors**		
Work-related physical activity (reference: Non-sitting)		
Sitting	1.93 (1.20, 3.09)	**0.007**
Distance live from work (reference: >5 miles)		
2.1-5 miles	2.04 (1.55, 2.68)	**<0.001**
≤2 miles	5.32 (3.85, 7.39)	**<0.001**
Free car parking at work (reference: Yes)		
No	3.73 (2.89, 4.81)	**<0.001**
Work hours (reference: Part-time)		
Full-time	1.06 (0.80, 1.40)	0.710
Work pattern (reference: Regular day time hours)		
Shift patterns	2.18 (1.46, 3.25)	**<0.001**
Occupation (reference: Manager/professional)		
Clerical or administrative	2.00 (1.57, 2.55)	**<0.001**

[a]OR (CI)=odds ratio (95% confidence interval)
