# Supplementary material for: Individual, employment and psychosocial factors influencing walking to work: Implications for intervention design
Source: PLoS One. 2017 Feb 9;12(2):e0171374. doi: 10.1371/journal.pone.0171374 (PMC5300108; doi:10.1371/journal.pone.0171374)
Supplement: S2 Table — (PDF) [file pone.0171374.s002.pdf]

Table S2. Univariate associations for perceived barriers, psychosocial factors and likelihood of commuter walking

	Commuter non-walkers Total n=602 n[a] (% agree)	Commuter walkers Total n=587 n[a] (% agree)	Univariate association with commuter walking OR (CI)[b]	p-value
**Perceived barriers[c]**				
I live too far away from work	403 (66.9)	210 (35.8)	0.28 (0.22, 0.35)	**<0.001**
Less convenient than using a car	296 (49.2)	84 (14.3)	0.17 (0.13, 0.23)	**<0.001**
It takes too long	289 (48.0)	149 (25.4)	0.37 (0.29, 0.47)	**<0.001**
I don't have time	249 (41.4)	126 (21.5)	0.39 (0.30, 0.50)	**<0.001**
I need a car to do my job	198 (32.9)	66 (11.2)	0.26 (0.19, 0.35)	**<0.001**
I have too much to carry	132 (21.9)	95 (16.2)	0.69 (0.51, 0.92)	**0.012**
Need to do other activities on the way	122 (20.3)	57 (9.7)	0.42 (0.30, 0.59)	**<0.001**
Need to drop off/collect children	121 (20.1)	63 (10.7)	0.48 (0.34, 0.66)	**<0.001**
I do lots of other activity/sport	91 (15.1)	61 (10.4)	0.65 (0.46, 0.92)	**0.015**
I've always travelled the same way	77 (12.8)	38 (6.5)	0.47 (0.31, 0.71)	**<0.001**
**Psychosocial factors**				
Attitude[d]	203 (37.9)	366 (70.0)	3.81 (2.95, 4.92)	**<0.001**
Perceived behavioural control[d]	152 (26.8)	481 (86.5)	17.55 (12.92, 23.84)	**<0.001**
Intention[d]	95 (16.9)	430 (77.8)	17.19 (12.76, 23.15)	**<0.001**
Social norm[d]	122 (24.5)	189 (40.8)	2.12 (1.61, 2.79)	**<0.001**
Social support from colleagues[e]	44 (7.5)	100 (17.7)	2.63 (1.81, 3.83)	**<0.001**

[a]Numbers do not sum to total due to missing responses
[b]OR (CI)=odds ratio (95% confidence interval)
[c]The reference category is no (item is not considered a barrier)
[d]The reference category is strongly disagree or disagree
[e]The reference category is disagree
